# Guidewire-assisted piecemeal resection of a giant gastric tumor

**DOI:** 10.1055/a-2218-3297

**Published:** 2024-01-09

**Authors:** Jixiang Ao, Silin Huang, Suhuan Liao, Longbin Huang, Erzhen Zhong, Pengcheng Zhao, Dongfang Wang

**Affiliations:** 1 Department of Gastroenterology, South China Hospital, Medical School, Shenzhen University, Shenzhen, China

A 55-year-old woman underwent esophagogastroduodenoscopy, which revealed a large subepithelial lesion located at the gastroesophageal junction, presenting a “horseshoe” morphology ( Fig. 1 ). A subsequent computed tomography (CT) scan and endoscopic ultrasonography indicated the lesion was a solid mass protruding into the lumen. The patient then underwent endoscopic submucosal resection, resulting in complete excision of the lesion. Due to the considerable size of the tumor, traditional snare-based extraction was unfeasible. Therefore, an innovative slicing technique was employed ( Video 1 ).

**Fig. 1:**
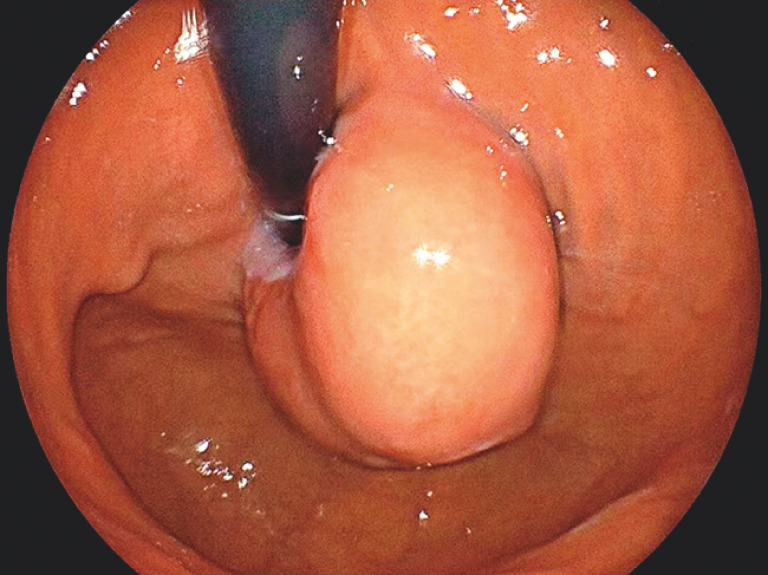
A large subepithelial lesion located at the gastroesophageal junction, presenting a “horseshoe” morphology.

Guidewire-assisted piecemeal resection of a giant gastric tumor.Video 1

To implement this technique, a transparent cap was affixed to the distal end of the endoscope. A guidewire, ingeniously shaped into a semicircle ( Fig. 2 ), was inserted into the working channel. Under direct visualization, the waist of the tumor was ensnared and repositioned anteriorly, aligned with the transparent cap. The guidewire was then meticulously retracted, enabling precise cold cutting of the tumor ( Fig. 3 ). This process was iteratively executed, allowing sequential removal of divided tumor fragments ( Fig. 4 ). Subsequent histopathological analysis confirmed a leiomyoma.

**Fig. 2:**
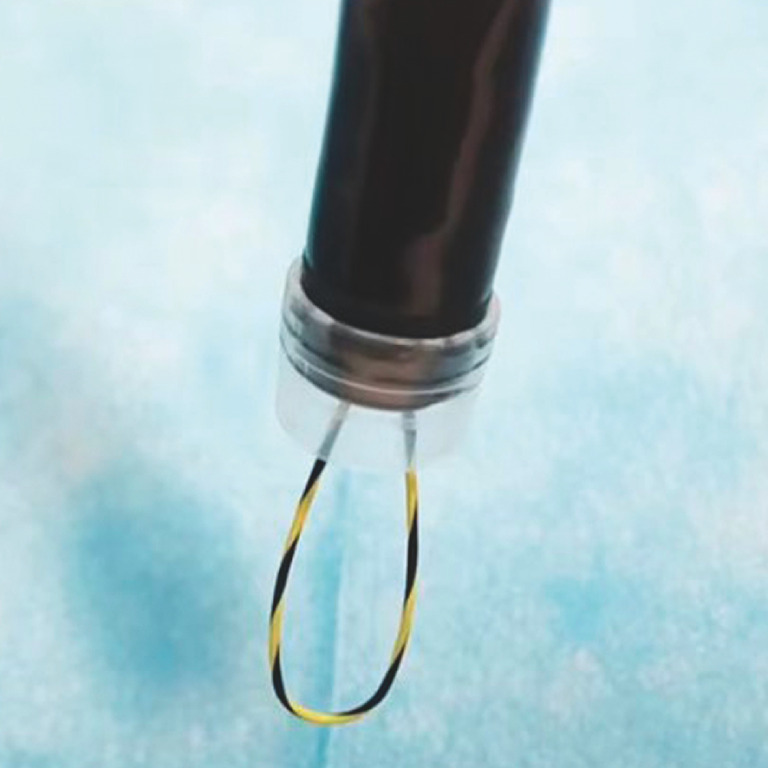
A new slicing device with guidewire.

**Fig. 3:**
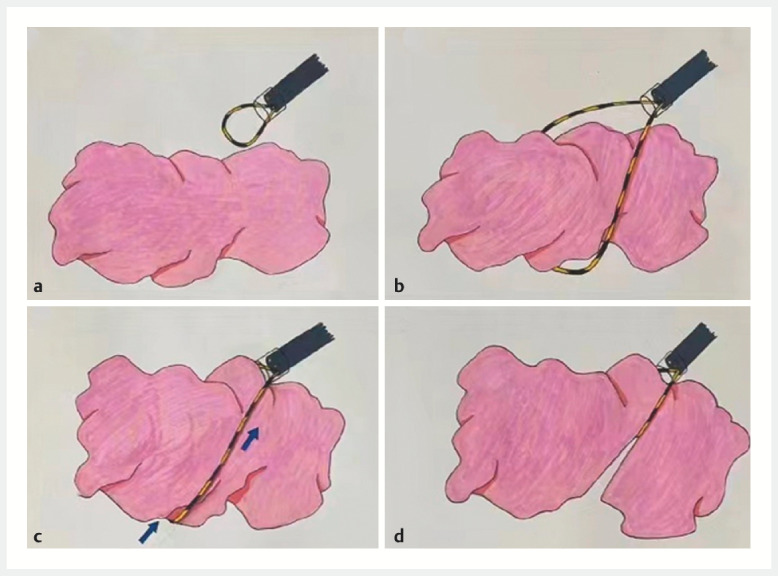
**a** Following endoscopic submucosal resection, a sizable mass was observed within the gastric cavity, necessitating the repositioning of the guidewire-assisted piecemeal resection device. **b** Within the confines of direct visual examination, the hemispherical guidewire, situated at the anterior endoscope, can be deftly adjusted to accommodate the tumorʼs encircled waist. **c** As the guidewire exterior to the endoscopeʼs working channel was grasped and drawn taut, the semicircular snare positioned at the forefront of the guidewire was gently maneuvered towards the anterior extremity of the transparent cap. **d** The tumors underwent an adroitly executed, precise cold cutting procedure, resulting in their methodical segmentation into distinct fragments.

**Fig. 4:**
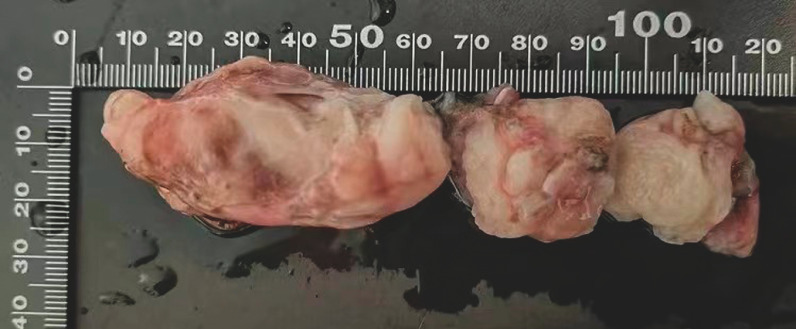
The specimen was methodically extracted in segmented portions, measuring approximately 10 centimeters in length.

This technique, while previously reported for disintegration of robust and oversized gastric bezoars 1 , had not found prior application for excision of sizable, non-extractable gastric masses. With the development of endoscopic excision techniques, comprehensive resection of enormous gastric leiomyomas is now achievable 2 3 . However, to prevent and manage post-resection intestinal obstruction from tumor migration and obtain definitive histopathological diagnosis, guidewire-assisted cold cutting emerges as an efficacious approach.

Endoscopy_UCTN_Code_CCL_1AB_2AD_3AF

## Notes

Endoscopy E-Videos https://eref.thieme.de/e-videos
*E-Videos* is an open access online section of the journal *Endoscopy* , reporting on interesting cases and new techniques in gastroenterological endoscopy. All papers include a high-quality video and are published with a Creative Commons CC-BY license. Endoscopy E-Videos qualify for HINARI discounts and waivers and eligibility is automatically checked during the submission process. We grant 100% waivers to articles whose corresponding authors are based in Group A countries and 50% waivers to those who are based in Group B countries as classified by Research4Life (see: https://www.research4life.org/access/eligibility/ ). This section has its own submission website at https://mc.manuscriptcentral.com/e-videos .

## References

[1] Hu X Zhang RY Liu WH A novel endoscopic treatment for giant gastric bezoars: guidewire-based seesaw-type fragmentation using a specific bezoaratom kit Endoscopy 2020 52 E146 E147 10.1055/a-0982-2661 31731317

[2] Jacobson BC Bhatt Amit Greer BK ACG clinical guideline: diagnosis and management of gastrointestinal subepithelial lesions Am J Gastroenterol 2023 118 46 58 36602835 10.14309/ajg.0000000000002100

[3] Ishita Dalal Iman Andalib Advances in endoscopic resection: a review of endoscopic submucosal dissection (ESD), endoscopic full thickness resection (EFTR) and submucosal tunneling endoscopic resection (STER) Transl Gastroenterol Hepatol 2022 7 19 10.21037/tgh-2020-10 35548477 PMC9081920

